# Advanced Studies in Clinical and Experimental Research in Gastroenterology

**DOI:** 10.1155/2016/4253143

**Published:** 2016-02-03

**Authors:** Ilja Tacheci, Jithinraj Edakkanambeth Varayil, Zuzana Zelinkova, Marcela Kopacova, Miroslav Zavoral

**Affiliations:** ^1^2nd Department of Internal Medicine-Gastroenterology, Charles University in Praha, Faculty of Medicine at Hradec Kralove, University Teaching Hospital, Sokolska 581, 500 05 Hradec Kralove, Czech Republic; ^2^Division of General Internal Medicine, Division of Gastroenterology and Hepatology, Mayo Clinic, Rochester, MN 55905, USA; ^3^5th Department of Internal Medicine, Comenius University in Bratislava, Faculty of Medicine in Bratislava, University Teaching Hospital, Ruzinovska 6, 826 06 Bratislava, Slovakia; ^4^Department of Internal Medicine, First Faculty of Medicine of Charles University and Military University Hospital, U Vojenské nemocnice 1200, 169 02 Prague, Czech Republic

Current research in gastroenterology and hepatology (both clinical and experimental) made great and rapid progress in new methods, in biomedical technology, and last but not least in practical application of basic scientific achievements during the last decade.

Original research articles focusing on new, advanced studies in clinical and experimental research in gastroenterology and hepatology that have a potential to influence our daily practice and further clinical and experimental research were sent to this journal to be published by many leading gastroenterology specialists.

We bring ten articles in this special issue focused on the most interesting topics in gastroenterology and hepatology research. One of the leading areas of advanced studies in gastroenterology is cancer research. Colorectal cancer is one of the most common malignancies worldwide in terms of incidence and mortality. Although clear progress in both diagnosis and treatment has been achieved, efficacy of surgery and chemotherapy remains unsatisfactory due to late diagnosis. A possible new prognostic marker and therapeutic target in this disease is discussed in the article “ATAD2 Overexpression Identifies Colorectal Cancer Patients with Poor Prognosis and Drives Proliferation of Cancer Cells.” The aim of another article dealing with the colorectal cancer “Molecular Features and Methylation Status in Early Onset (≤40 Years) Colorectal Cancer: A Population Based, Case-Control Study” was to define the frequency of known hereditary colorectal syndromes and to characterise genetic and epigenetic features of early nonhereditary tumours in order to elucidate possible pathogenetic mechanisms. Colonoscopy plays an important role in colorectal cancer screening in many countries worldwide. Quality of colonic cleansing achieved is crucial for good quality of colonoscopy screening. Possible improvements in the field of low volume colonoscopy cleansing were described in the article “A Randomized Controlled Trial Evaluating a Low-Volume PEG Solution Plus Ascorbic Acid versus Standard PEG Solution in Bowel Preparation for Colonoscopy.” Second malignancy, which is discussed in a special issue in more detail, is the third most common cause of cancer-related death in the world: the gastric cancer. In the article “The Prevalence of Gastric Intestinal Metaplasia and Distribution of* Helicobacter pylori* Infection, Atrophy, Dysplasia, and Cancer in Its Subtypes,” the prevalence of the precancerous gastric lesions (intestinal metaplasia especially) in a large cohort of patients within the region with a high risk of gastric cancer was described. The metastatic involvement of the posterior lymph nodes along the common hepatic artery (the extra-regional lymph nodes called No.8p) was identified as a prognostic factor in the retrospective study: “Prognostic Value of Metastatic No.8p LNs in Patients with Gastric Cancer.”

The research based on the use of experimental animals still plays an important role in gastroenterology and the rat is one of the most extensively used experimental animal models.

The study “Surgical Anatomy of the Gastrointestinal Tract and Its Vasculature in the Laboratory Rat” investigated the functional anatomy and vasculature of the stomach, liver, and intestine in the laboratory rat and compared it with human morphology.

Nonsteroidal anti-inflammatory drug-induced gastropathy and ulcers represent an important complication related to one of the most commonly used drugs worldwide. Possible mucosal protective effect of* Syzygium cumini* (L.) Skeels aqueous extract in experimental mice model of indomethacin-induced gastric damage was proven in article “Anti-Inflammation Property of* Syzygium cumini* (L.) Skeels on Indomethacin-Induced Acute Gastric Ulceration.”

Research in hepatology is represented in three articles: “Assessing the Effect of Leptin on Liver Damage in Case of Hepatic Injury Associated with Paracetamol Poisoning,” “Lower Viral Response to Pegylated Interferon Alpha 2a Treatment of Chronic Hepatitis B in Roma People in Eastern Slovakia,” and “Serum Liver Fibrosis Markers for Predicting the Presence of Gastroesophageal Varices in Liver Cirrhosis: A Retrospective Cross-Sectional Study.”

The special issue cannot be a complete overview of research in the field of gastroenterology and hepatology. However, we believe that it is an interesting look at this area and provides a preview with an important impact on clinical medicine.

*Ilja Tacheci* *Ilja Tacheci* *Jithinraj Edakkanambeth Varayil* *Jithinraj Edakkanambeth Varayil* *Zuzana Zelinkova* *Zuzana Zelinkova* *Marcela Kopacova* *Marcela Kopacova* *Miroslav Zavoral* *Miroslav Zavoral*

